# Spontaneous Zero-Field Cooling Exchange Bias in Ni–Co–Mn–Sn Metamagnetic Heusler Sputtered Film

**DOI:** 10.3390/nano11092188

**Published:** 2021-08-26

**Authors:** Vasileios Alexandrakis, Iván Rodríguez-Aseguinolaza, Dimitrios Anastasakos-Paraskevopoulos, Jose Manuel Barandiarán, Volodymyr Chernenko, Jose Maria Porro

**Affiliations:** 1 Institute of Nanoscience and Nanotechology, National Center for Scientific Research “Demokritos”, 15310 Agia Paraskevi, Greece; d.anastasakos@inn.demokritos.gr; 2 Department of Electricity & Electronics, University of the Basque Country (UPV/EHU), 48080 Bilbao, Spain; irodriguez037@gmail.com (I.R.-A.); josemanuel.barandiaran@ehu.eus (J.M.B.); volodymyr.chernenko@bcmaterials.net (V.C.); 3 Basque Center for Materials, Applications and Nanostructures “BCMaterials”, 48940 Leioa, Spain; 4 Ikerbasque—Basque Foundation for Science, 48009 Bilbao, Spain

**Keywords:** metamagnetic Heusler alloys, spontaneous exchange bias, AC susceptibility, superferromagnetism

## Abstract

Metamagnetic off-stoichiometric Heusler alloys are actively being investigated because of their great potential as magnetocaloric materials. These properties are intimately related to the nanoscale homogeneity of their magnetic properties, mainly due to a strong influence of the nature of the exchange interactions between Mn atoms on the magnetism of the alloys. In this work, a spontaneous exchange bias phenomenon on a Ni–Co–Mn–Sn metamagnetic Heusler sputtered film is presented and studied in detail. More particularly, a series of DC magnetization curves measured as a function of the temperature demonstrates that the system exhibits canonical spin glass-like features. After a careful study of the field-cooling and zero-field-cooling curves measured on this system, the existence of magnetic inhomogeneities is inferred, as a consequence of the competition between ferromagnetic and antiferromagnetic exchange interactions between Mn atoms. Further AC susceptibility measurements on this system demonstrate that the underlying exchange bias phenomenon can be attributed to a magnetic clusters model based on superferromagnetic-like interactions present in the film. These findings suggest that the spontaneous exchange bias exhibited by the studied system is a consequence of the formation of this superferromagnetic-like state.

## 1. Introduction

Recently, Ni_50_Mn_25+x_Sn_25−x_ metamagnetic off-stoichiometric Heusler alloys (X_2_YZ, where X = Ni, Y = Mn and Z = Sn) have attracted the attention of the scientific community due to their magnetocaloric properties [1,2,3,4] which are closely related to the nanoscale magnetic homogeneity [5,6,7,8]. The latter is caused by the sensitivity of Mn exchange interactions to interatomic distances and local symmetry [9,10,11,12,13].

This complicated magnetic state promotes exchange bias phenomena (EB) in metamagnetic Heusler alloys [14,15]. It is known that exchange bias is attributed to spin configuration between antiferromagnetic/ferromagnetic (AF/FM) interfaces [16,17,18], ferromagnetic/spin glass [19] or AF/ferromagnetic [20] interfaces and it is very common in nanostructures [21,22] or thin film bilayers [23,24,25]. The most common applications of exchange bias are in rigid and flexible spintronic devices [26,27,28,29,30] (i.e., magnetic tunnelling junctions, Magnetoresistive Random-Access Memory) but recently AF/FM nanocomposites, based on NiO/Ni, were also proposed as efficient microwave absorbers [31]. Therefore, it is not expected in bulk materials where there are no interfaces and the surface to volume ratio is very low. But in off-stoichiometric metamagnetic Heusler alloys, EB occurs in bulk polycrystalline samples [32,33,34] due to the nanoscale magnetic inhomogeneity.

It is evident that the observation of EB in bulk off-stoichiometric Heusler alloys is a peculiar case of EB, but even more intriguing is the existence of spontaneous exchange bias after zero-field cooling (ZFC) [35,36,37,38]. The difference between common EB and ZFC-EB is that the latter takes place without the application of an external magnetic field during cooling process. Particularly, EB in common AF/FM composites does not occur without the influence of an applied magnetic field during the cooling process from high temperatures below the Néel temperature. Contrarily, in ZFC-EB phenomena no applied field is necessary during the cooling process for the occurrence of EB. According to the existing models, ZFC-EB, in off-stoichiometric Heusler alloys, is correlated to short range magnetic interactions [35,38,39], e.g., super ferromagnetism or super spin glass state. In the present study, we investigate FC and ZFC exchange bias in an epitaxial Ni–Co–Mn–Sn sputtered film and we probe, for first time, the evolution of a superferromagnetic-like state using AC susceptibility measurements at different DC bias fields.

## 2. Materials and Methods

A Ni–Co–Mn–Sn film was sputtered by a Ni–Co–Mn–Sn alloy target on a cubic MgO (100) substrate at 773 K using a Pfeiffer Vacuum Classic 500 sputtering system. The Ar deposition pressure was 2.6 × 10^−2^ mbar and the thickness of the deposited film was 1 μm. The composition of the film was determined by energy-dispersive X-ray spectroscopy (EDX) analysis using a built-in EDX system in a Hitachi TM-3000 scanning electron microscope. X-ray diffraction (XRD) measurements, using a Bruker D8 Advance Vantec diffractometer equipped with a HTK 2000 temperature chamber and an ultrarapid area detector with a maximum aperture of 6°, confirmed the epitaxiality of the deposited Ni–Co–Mn–Sn alloy film. Further details about the film growth can be found in [40]. All magnetic measurements, including DC magnetometry, ZFC-FC measurements and AC susceptibility measurements were performed in a Magnetic Property Measurement System (MPMS) equipped with a superconducting magnet, using a Quantum Design MPMS-3 SQUID magnetometer. Details on the sequences employed to perform the magnetic measurements are available on reasonable demand from the corresponding authors.

## 3. Results and Discussion

The 500 nm thick Ni–Co–Mn–Sn epitaxial film, issue of study in the present manuscript, was grown by sputtering deposition on top of a cubic MgO substrate at high temperature. The epitaxiality of the sample was confirmed by means of X-ray diffraction measurements, showing a unit cell rotated 45° with respect to that of the substrate: Ni–Co–Mn–Sn (001) [110] || MgO (001) [100]. Further details about the film growth and structural characterization can be found in [40]. The composition of the film was determined by energy-dispersive X-ray spectroscopy (EDX) analysis and found to be Ni_47_Co_6_Mn_35_Sn_12_.

**Figure 1:**
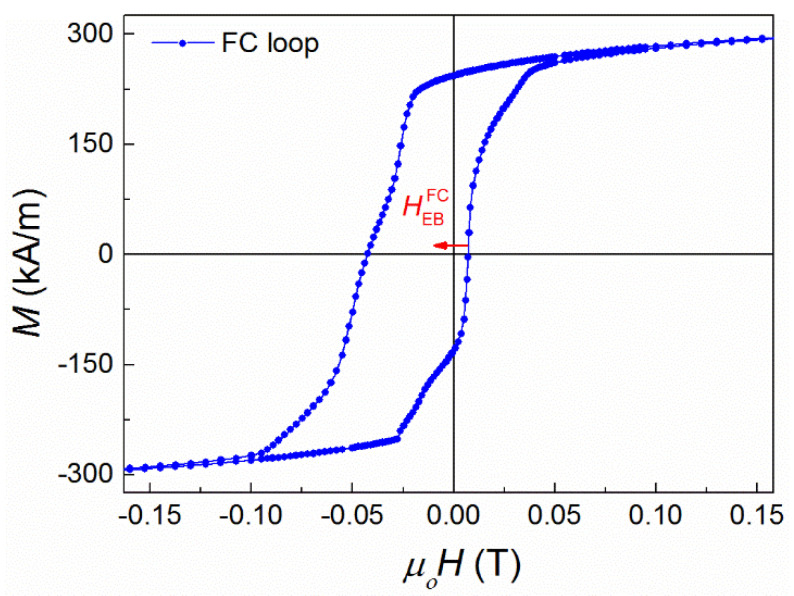
Field cooling (FC) hysteresis loop of the Ni–Co–Mn–Sn film at 5 K, cooled from 400 K under the influence of a cooling field of 1 T.

The field cooling (FC) hysteresis loop (Figure 1) was recorded at 5 K after cooling down under a field of 1 T from 400 K, well above Tc of the austenite phase, where the sample is in its paramagnetic phase. It should be stressed that for the zero-field cooling (ZFC) measurements, in order to achieve real and accurate zero-field conditions during the cooling process from 400 K to 5 K, the superconducting magnet was quenched to eliminate any residual superconducting currents, which could result in a small residual field during the ZFC measurements. Finally, the maximum sweeping field of the hysteresis loop at 5 K was 2 T. The FC loop was shifted along the negative field axis, thus indicating the existence of exchange bias, with an exchange bias field *μ_ο_*$H_{EB}^{FC}$ = 0.0175 T. It is known that EB is a phenomenon which develops at the interfaces between an AF and a FM region, thereby requiring the coexistence of AF and FM phases (or alternatively a spin glass state), which, in the case of a single-phase Ni–Co–Mn–Sn Heusler alloy, can be realized through the formation of AF and FM domains created by the interplay between FM and AF interactions due to the anti-site disorder. Since there are no indications of the formation of a secondary structural phase, we conclude that both magnetic phases, AF and FM, coexist as magnetic domains in the same structural substance (Ni–Co–Mn–Sn alloy).

**Figure 2:**
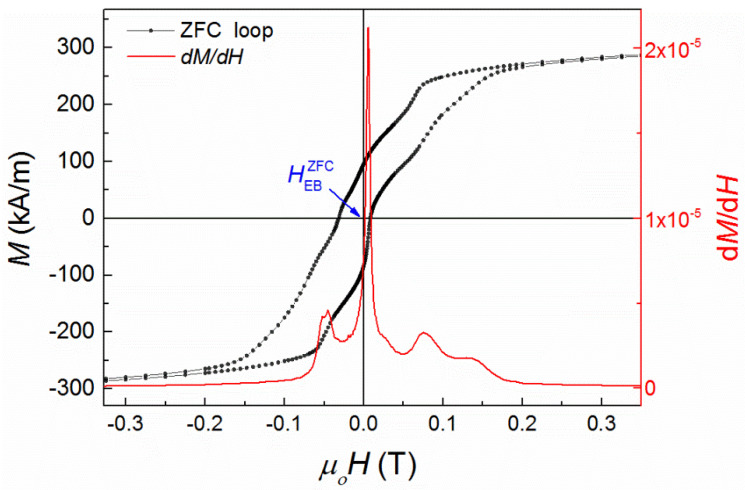
Zero-field cooling (ZFC) loop (black curve) at 5 K, after quenching the superconducting magnet, and the derivative of the magnetization as a function of the applied field calculated in the second half of the loop (−2 T to 2 T; red curve).

Indeed, as it has already been mentioned in the introduction, when Sn sites (Z sublattice in X_2_YZ notation of a stoichiometric full Heusler alloy) are occupied by Mn (anti-site disorder), then antiferromagnetic coupling is induced between adjacent Mn atoms in Y and Z sublattices (Mn_z_–Mn_y_). Actually, a Ruderman–Kittel–Kasuya–Yosida-like (RKKY-like) oscillating exchange interaction has been identified between Mn atoms in off-stoichiometric Heusler alloys [7], which results in alternating positive (ferromagnetic) and negative (antiferromagnetic) coupling as a function of the Mn_z_–Mn_y_ interatomic distance. Furthermore, at low temperatures the system undergoes a martensitic structural transformation from a high symmetry (cubic) austenite phase to a low symmetry (orthorhombic) martensitic phase [40], which varies further the interatomic distances of Mn along different crystallographic directions, increasing the competition between FM and AF interactions of Mn magnetic moments. The presence of competing magnetic interactions, in a low symmetry structure, leads to a complicated free energy landscape having several local minima separated by energy barriers that grow exponentially. Therefore, the interplay between AF and FM interactions results in the formation of a metastable domain state consisting of a mixture of FM and AF domain regions. Indeed, the ZFC hysteresis loop recorded at 5 K, shown in Figure 2, verifies this picture, since the magnetization switching seems to take place in several stages, indicated by the local minima of the first derivative of magnetization (Figure 2), suggesting a domain wall pinning mechanism.

**Figure 3:**
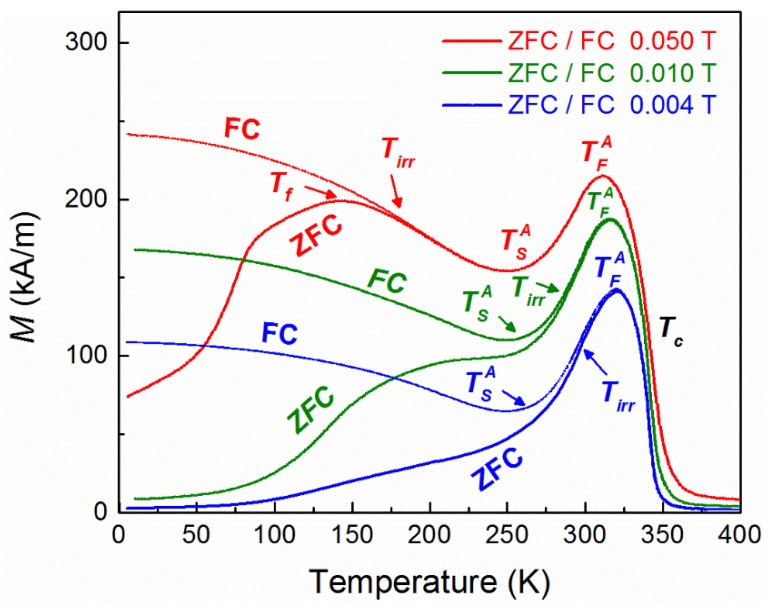
Zero-field cooling and field cooling magnetization curves for various cooling fields.

Except the unusual magnetization reversal mechanism, the system exhibits also spontaneous exchange bias at 5 K, *μ_ο_*$H_{EB}^{ZFC}$ = 0.0117 T (as extracted from the measurements shown in Figure 2), after zero-field cooling. In order to obtain further insights into the mechanism of ZFC exchange bias, FC and ZFC magnetization curves and AC susceptibility measurements as a function of temperature were performed. Particularly, the features above 220 K, in FC magnetization curves, are related to the martensitic–austenitic magnetostructural transformation (austenitic start $\left(T_{S}^{A}\sim \;240\;K\right)$ and austenitic finish $\left(T_{F}^{A}\sim \;310\;K\right)$ temperatures) and the Curie point (*T* > 310 K). Below 200 K, zero-field cooling and field cooling magnetization curves (Figure 3) show a similar behavior to that observed in a classical spin glass system. It is known that in a canonical spin glass system, the bifurcation point (*T_irr_*) between FC and ZFC is very close to the local maximum (*T_f_*) or plateau of the ZFC curve. In our system, there is significant deviation between *T_irr_* and *T_f_*, especially for low fields (<10 mT), which indicates that the system is not magnetically homogeneous [41] although the material is compositionally homogeneous. This may indicate the existence of a magnetic cluster phase [42] in the sample. The magnetic inhomogeneity could be attributed to the interplay between ferromagnetic and antiferromagnetic interactions of Mn, resulting in short range magnetic (FM or AF) ordering.

**Figure 4:**
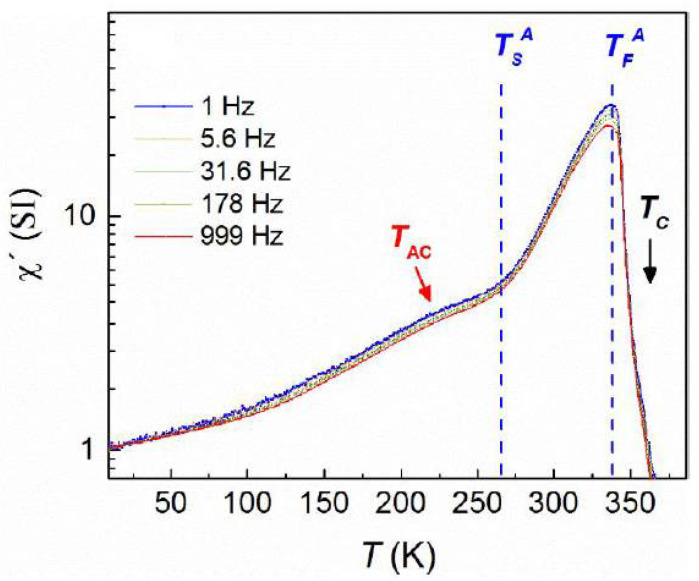
Log scale plot of the AC volume susceptibility after ZFC at zero DC bias field and different frequencies *f*. Units are SI.

It is well known that the temperature dependence of the AC susceptibility peak provides significant information about the nature of the magnetic interactions of a glassy system. For this reason, AC measurements at various frequencies (*f*) were carried out in zero (DC) field cooling mode, starting from 400 K, which is above T_c_ of the austenite phase. The AC field was 0.2 mT for all measurements. The real part of the AC susceptibility (Figure 4) showed the same features as those of ΖFC DC magnetization measurements recorded with a heating ramp at 0.004 T (Figure 3). Particularly the asymmetric peak around 336 K indicates the martensitic transition (335 K–265 K), which is followed, at higher temperatures (T > 336 K), by the paramagnetic transition of the austenite phase at the Curie temperature (T_c_ = 350 K). At lower temperatures, there is a very broad peak around 220 K (*T_AC_*) which decays towards 5 K (Figure 4) and corresponds to the ZFC curve of the DC magnetization measurements at 0.004 T shown in Figure 3. Actually, this peak displaces to higher temperatures with increasing frequency. The dependence of the relaxation times on the peak temperatures exhibits an exponential behavior, but fittings to both Néel–Arrhenius and Vogel–Fulcher–Tammann models, for non-interacting or weakly interacting particles, respectively, yield unphysical values.

**Figure 5:**
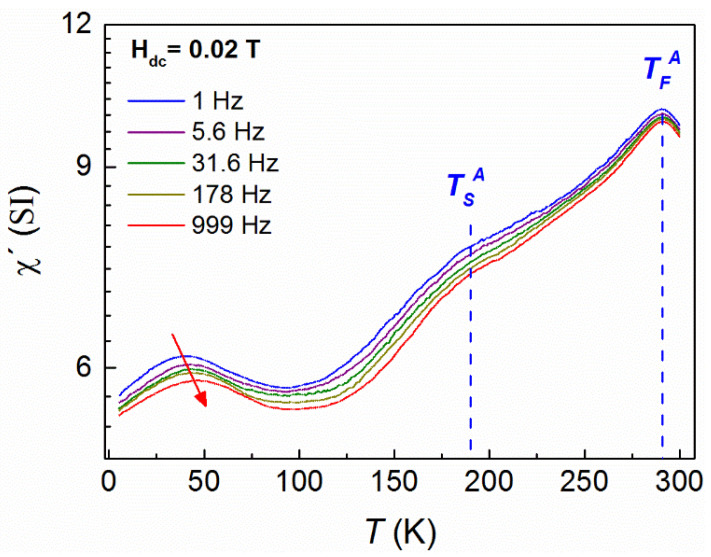
Log scale plots of the AC susceptibility measurements taken after ZFC, at 0.02 T DC bias field.

To clarify the situation, AC measurements were repeated, using the same conditions as in ZFC AC measurements, but this time a DC bias field of 0.0200 T was applied at 5 K after ZFC. Then, an additional peak was observed around 42 K which did not exist in the initial ZFC measurements at zero DC bias field (Figure 5), indicating the formation of an emerging magnetic state. The peak also shifts towards higher temperatures as frequency increases; this is a fingerprint of a magnetization relaxation process. As previously mentioned for the zero-field cooling DC bias measurements, the analysis of the experimental data using either Néel–Arrhenius or Vogel–Fulcher–Tammann models, for non-interacting or weakly interacting particles, respectively, does not give reasonable results, therefore another model should be used.

The weak shift of the AC susceptibility peak, when a DC bias field is applied, is a sign of critical slowing down of the magnetization relaxation as the system approaches a phase transition at the temperature T_g_ [43]. The nonanalytic behavior at T_g_ results from the growth of correlations among the spins as T_g_ is approached. The conventional approach of dynamical scaling relates the relaxation time τ for the decay of the fluctuations to the spin correlation length ξ as τ ~ ξ^Z^, where z is the dynamical critical exponent [44,45]. Since the spin correlation length, near the phase transition, diverges with temperature as [(T − T_g_)/T_g_]^−v^, where v is a critical exponent, the expression for τ is given by:

(1) $$\tau \;=\;\tau_{o}\;{\left(\frac{T}{T_{g}}-1\right)}^{-\mathrm{zv}}$$

where T_g_ is the static (*f* tends to zero) spin glass temperature which marks the onset of critical slowing and collective glassy behavior; zν is the dynamical critical exponent which is related to the correlation length ξ that diverges at Tg. The abovementioned phenomenological activation law is usually employed for spin cluster magnetic systems.

Τhe fitting parameters based on this specific model yield relatively reasonable values, even though the range of frequencies was limited to 1 kHz. Based on this model, the glass transition temperature T_g_ is 37 K, the critical exponent is 9.37 and the relaxation time is 2.6 × 10^−9^ s. Similar values have been found in Ni–Mn–In compounds [30]. The latter seems to be high compared to canonical spin glass systems, where τ is determined by the spin flip time of the atomic magnetic moments (10^−13^ s), but in a cluster system the characteristic relaxation time is determined by the spin flip time of the clusters, which is always higher. Ιn the present study, magnetic interactions take place mainly through exchange interactions—since the magnetic clusters are accommodated in either the same crystallites or neighboring crystallites which are in contact—which are always stronger than dipolar ones; therefore, a high relaxation time is very likely. Consequently, the first indications suggest that the system is well described by the cluster model.

**Figure 6:**
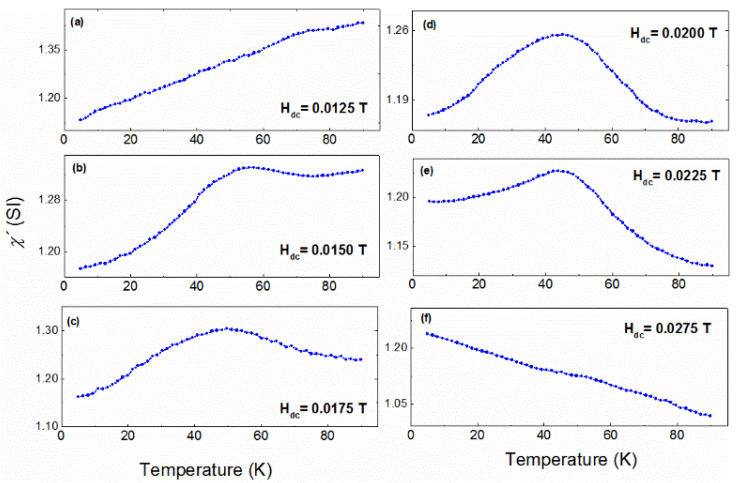
Evolution of the AC susceptibility peak close to 42 K after ZFC, as a function of temperature for different DC bias fields (H_dc_) at 178 Hz; (**a**) H_dc_ = 0.0125 T, (**b**) H_dc_ = 0.0150 T, (**c**) H_dc_ = 0.0175 T, (**d**) H_dc_ = 0.0200 T, (**e**) H_dc_ = 0.0225 T, (**f**) H_dc_ = 0.0275 T.

To verify this conclusion, similar AC susceptibility measurements were performed at a fixed frequency of 178 Hz while varying the DC bias field (0.0050 T < H_DC_ < 0.0350 T). The aim of these measurements is to manipulate the size of the clusters and check its influence on the AC susceptibility. Indeed, as observed in Figure 6, the peak of the AC susceptibility measurements, around 42 K, progressively increases as the DC field increases from 0.0100 T to 0.0200 T and is also progressively decomposed at higher fields from 0.0200 T to 0.0350 T.

The evolution of this specific peak as a function of the DC bias field verifies the existence of clusters and gives an insight into the interactions between them; when the external DC bias field reaches a critical value, collective interactions between the magnetic clusters develop, giving rise to a maximum in the AC susceptibility. Above this critical value, the Zeeman energy dominates, forcing the magnetic moments to align parallel to the direction of the field and forcing the response of AC susceptibility to be linear with the external field. Usually, in an ensemble of nanoparticles, a superferromagnetic (SF) state is achieved when the system is close to the percolation limit, when most of the particles are very close to each other and strong interparticle interactions develop (i.e., dipolar interactions). In this metamagnetic Heusler alloy in the form of dense films, the magnetic nanoparticles have been replaced by nanoscale magnetic domains and a SF state is realized through the expansion of the individual magnetic domains. In our case, the critical field seems to be close to 0.02 T, as indicated by the AC susceptibility measurements, performed at various DC fields (Figure 6). Indeed, at 0.02 T the observed peak presents its maximum height, relative to the other peaks, at different fields.

All these findings verify the proposed exchange bias model based on the formation of a cluster superferromagnetic-like state for the interpretation of the spontaneous exchange bias in metamagnetic Heusler alloys. Particularly, the unstable ferromagnetic domains (as indicated by ZFC–FC magnetization measurements presented in Figure 3) grow progressively with increasing external magnetic field after ZFC. Then, collective interactions are induced through superexchange when the critical field is reached, forming a superferromagnetic-like (SF) state with a unidirectional magnetic moment. Since the SF clusters coexist with antiferromagnetic domains (due to Mn–Mn interactions), a new stable interface is formed consisting of the domains of the superferromagnetic state and the antiferromagnetic ones. This phenomenon results in pinning of the magnetic moments of the superferromagnetic-like state, below the blocking temperature, in a similar manner to exchange bias in conventional ferromagnetic/antiferromagnetic interfaces.

## 4. Conclusions

To sum up, a detailed study of spontaneous exchange bias in metamagnetic Heusler alloys has been carried out. Particularly, DC magnetization curves as a function of temperature showed that the system exhibits similar features to those of a canonical spin glass system. However, the divergence between the bifurcation point of FC and ZFC curves and the local maximum or plateau of the ZFC curve suggests the existence of magnetic inhomogeneities, which is related to the competition between ferromagnetic and antiferromagnetic interactions of Mn atoms. To clarify the situation, we performed, to our knowledge, a first-time study consisting of ZFC AC susceptibility measurements at different constant DC bias fields. Then, a new peak arose at low temperatures (Figure 5), which has not been observed in common ZFC AC susceptibility measurements without a DC bias field (Figure 4). It is known that the existence of peaks in AC susceptibility measurements is related to the formation of a new magnetic state. Indeed, a mathematical analysis showed that the results are described by a magnetic clusters model based on superferromagnetic-like interactions. Moreover, the evolution of the emerging superferromagnetic-like state was probed by varying the DC bias field in AC susceptibility measurements at a fixed frequency. All the aforementioned findings suggest that spontaneous exchange bias is related to the formation of a superferromagnetic-like state.

## Data Availability

Data available on request.

## References

[1] Gottschall T., Skokov K.P., Fries M., Taubel A., Radulov I., Scheibel F., Benke D., Riegg S., Gutfleisch O. (2019). Making a Cool Choice: The Materials Library of Magnetic Refrigeration. Adv. Energy Mater..

[2] Liu J., Gottschall T., Skokov K.P., Moore J.D., Gutfleisch O. (2012). Giant Magnetocaloric Effect Driven by Structural Transitions. Nat. Mater..

[3] Zhang K., Tan C., Zhao W., Guo E., Tian X. (2019). Computation-Guided Design of Ni–Mn–Sn Ferromagnetic Shape Memory Alloy with Giant Magnetocaloric Effect and Excellent Mechanical Properties and High Working Temperature via Multielement Doping. ACS Appl. Mater. Interfaces.

[4] Gràcia-Condal A., Gottschall T., Pfeuffer L., Gutfleisch O., Planes A., Mañosa L. (2020). Multicaloric Effects in Metamagnetic Heusler Ni-Mn-In under Uniaxial Stress and Magnetic Field. Appl. Phys. Rev..

[5] Sokolovskiy V.V., Entel P., Buchelnikov V.D., Gruner M.E. (2015). Achieving Large Magnetocaloric Effects in Co- and Cr-Substituted Heusler Alloys: Predictions from First-Principles and Monte Carlo Studies. Phys. Rev. B Condens. Matter Mater. Phys..

[6] Entel P., Siewert M., Gruner M.E., Herper H.C., Comtesse D., Arróyave R., Singh N., Talapatra A., Sokolovskiy V.V., Buchelnikov V.D. (2013). Complex Magnetic Ordering as a Driving Mechanism of Multifunctional Properties of Heusler Alloys from First Principles. Eur. Phys. J. B.

[7] Comtesse D., Gruner M.E., Ogura M., Sokolovskiy V.V., Buchelnikov V.D., Grünebohm A., Arróyave R., Singh N., Gottschall T., Gutfleisch O. (2014). First-Principles Calculation of the Instability Leading to Giant Inverse Magnetocaloric Effects. Phys. Rev. B.

[8] Zhang L., Zhang J., Li K., Zhou C., Yao Y., Tan T.T., Wang D., Yang S., Li S., Carpenter M.A. (2020). Glassy Magnetic Transitions and Accurate Estimation of Magnetocaloric Effect in Ni–Mn Heusler Alloys. ACS Appl. Mater. Interfaces.

[9] Lázpita P., Barandiarán J.M., Gutiérrez J., Feuchtwanger J., Chernenko V.A., Richard M.L. (2011). Magnetic Moment and Chemical Order in Off-Stoichiometric Ni-Mn-Ga Ferromagnetic Shape Memory Alloys. New J. Phys..

[10] Richard M.L., Feuchtwanger J., Allen S.M., O’Handley R.C., Lázpita P., Barandiaran J.M., Gutierrez J., Ouladdiaf B., Mondelli C., Lograsso T. (2007). Chemical Order in Off-Stoichiometric Ni-Mn-Ga Ferromagnetic Shape-Memory Alloys Studied with Neutron Diffraction. Philos. Mag..

[11] Lázpita P., Barandiarán J.M., Gutiérrez J., Mondelli C., Sozinov A., Chernenko V.A. (2017). Polarized Neutron Study of Ni-Mn-Ga Alloys: Site-Specific Spin Density Affected by Martensitic Transformation. Phys. Rev. Lett..

[12] Golub V., L’vov V.A., Salyuk O., Barandiaran J.M., Chernenko V.A. (2020). Magnetism of Nanotwinned Martensite in Magnetic Shape Memory Alloys. J. Phys. Condens. Matter.

[13] Golub V., Aseguinolaza I.R., Salyuk O., Popadiuk D., Sharay I., Fernández R., Alexandrakis V., Bunyaev S.A., Kakazei G.N., Barandiarán J.M. (2021). Thickness Dependences of Structural and Magnetic Properties of Ni(Co)MnSn/MgO(001) Thin Films. J. Alloys Compd..

[14] Aksoy S., Acet M., Deen P.P., Mañosa L., Planes A. (2009). Magnetic Correlations in Martensitic Ni-Mn-Based Heusler Shape-Memory Alloys: Neutron Polarization Analysis. Phys. Rev. B Condens. Matter Mater. Phys..

[15] El-Khatib S., Bhatti K.P., Srivastava V., James R.D., Leighton C. (2019). Nanoscale Magnetic Phase Competition throughout the N I50-x C Ox M N40 S N10 Phase Diagram: Insights from Small-Angle Neutron Scattering. Phys. Rev. Mater..

[16] Meiklejohn W.H., Bean C.P. (1956). New Magnetic Anisotropy. Phys. Rev..

[17] Nogués J., Schuller I.K. (1999). Exchange Bias. J. Magn. Magn. Mater..

[18] Radu F., Zabel H., Zabel H., Bader S.D. (2008). Exchange Bias Effect of Ferro-/Antiferromagnetic Heterostructures. Magnetic Heterostructures: Advances and Perspectives in Spinstructures and Spintransport.

[19] Ali M., Adie P., Marrows C.H., Greig D., Hickey B.J., Stamps R.L. (2007). Exchange Bias Using a Spin Glass. Nat. Mater..

[20] Shell C., Mn M., Salazar-Alvarez G., Sort J., Suriñach S., Baro M.D., Nogués J. (2007). Synthesis and Size-Dependent Exchange Bias in Inverted Core−Shell MnO|Mn_3_O_4_ Nanoparticles. J. Am. Chem. Soc..

[21] Phan M.H., Alonso J., Khurshid H., Lampen-Kelley P., Chandra S., Repa K.S., Nemati Z., Das R., Iglesias Ó., Srikanth H. (2016). Exchange Bias Effects in Iron Oxide-Based Nanoparticle Systems. Nanomaterials.

[22] Markovich V., Puzniak R., Mogilyansky D., Wu X., Suzuki K., Fita I., Wisniewski A., Chen S., Gorodetsky G. (2011). Exchange Bias Effect in La0.2Ca0.8MnO3 Antiferromagnetic Nanoparticles with Two Ferromagnetic-like Contributions. J. Phys. Chem. C.

[23] Sharma A., Tripathi J., Tripathi S., Kumar Y., Ugochukwu K.C., Kumar D., Gupta M., Chaudhary R.J. (2020). Exchange Bias in Co/CoO Thin Films Deposited onto Self-Assembled Nanosphere Arrays. J. Magn. Magn. Mater..

[24] Stamps R.L. (2000). Mechanisms for Exchange Bias. J. Phys. D Appl. Phys..

[25] Liu K., Baker S.M., Tuominen M., Russell T.P., Schuller I.K. (2001). Tailoring Exchange Bias with Magnetic Nanostructures. Phys. Rev. B Condens. Matter Mater. Phys..

[26] Parkin S.S.P., Roche K.P., Samant M.G., Rice P.M., Beyers R.B., Scheuerlein R.E., O’Sullivan E.J., Brown S.L., Bucchigano J., Abraham D.W. (1999). Exchange-Biased Magnetic Tunnel Junctions and Application to Nonvolatile Magnetic Random Access Memory (Invited). J. Appl. Phys..

[27] Parkin S.S.P., Kaiser C., Panchula A., Rice P.M., Hughes B., Samant M., Yang S.H. (2004). Giant Tunnelling Magnetoresistance at Room Temperature with MgO (100) Tunnel Barriers. Nat. Mater..

[28] Zhang Z., Liu E., Zhang W., Wong P.K.J., Xu Z., Hu F., Li X., Tang J., Wee A.T.S., Xu F. (2019). Mechanical Strain Manipulation of Exchange Bias Field and Spin Dynamics in FeCo/IrMn Multilayers Grown on Flexible Substrates. ACS Appl. Mater. Interfaces.

[29] Huang J., Wang H., Wang X., Gao X., Liu J., Wang H. (2020). Exchange Bias in a La_0.67_Sr_0.33_MnO_3_/NiO Heterointerface Integrated on a Flexible Mica Substrate. ACS Appl. Mater. Interfaces.

[30] Perzanowski M., Zarzycki A., Gregor-Pawlowski J., Marszalek M. (2020). Magnetization Reversal Mechanism in Exchange-Biased Spring-like Thin-Film Composite. ACS Appl. Mater. Interfaces.

[31] You W., Che R. (2018). Excellent NiO-Ni Nanoplate Microwave Absorber via Pinning Effect of Antiferromagnetic-Ferromagnetic Interface. ACS Appl. Mater. Interfaces.

[32] Khan M., Dubenko I., Stadler S., Ali N. (2007). Exchange Bias Behavior in Ni-Mn-Sb Heusler Alloys. Appl. Phys. Lett..

[33] Li Z., Jing C., Chen J., Yuan S., Cao S., Zhang J. (2007). Observation of Exchange Bias in the Martensitic State of Ni50Mn36Sn14 Heusler Alloy. Appl. Phys. Lett..

[34] Khan M., Dubenko I., Stadler S., Ali N. (2007). Exchange Bias in Bulk Mn Rich Ni-Mn-Sn Heusler Alloys. J. Appl. Phys..

[35] Wang B.M., Liu Y., Ren P., Xia B., Ruan K.B., Yi J.B., Ding J., Li X.G., Wang L. (2011). Large Exchange Bias after Zero-Field Cooling from an Unmagnetized State. Phys. Rev. Lett..

[36] Wang B.M., Liu Y., Xia B., Ren P., Wang L. (2012). Large Exchange Bias Obtainable through Zero-Field Cooling from an Unmagnetized State in Ni-Mn-Sn Alloys. J. Appl. Phys..

[37] Liao P., Jing C., Wang X.L., Yang Y.J., Zheng D., Li Z., Kang B.J., Deng D.M., Cao S.X., Zhang J.C. (2014). Strongly Enhanced Antiferromagnetism and Giant Spontaneous Exchange Bias in Ni50Mn36Co4Sn10 Heusler Alloy. Appl. Phys. Lett..

[38] Nayak A.K., Nicklas M., Chadov S., Shekhar C., Skourski Y., Winterlik J., Felser C. (2013). Large Zero-Field Cooled Exchange-Bias in Bulk Mn_2_PtGa. Phys. Rev. Lett..

[39] Jia L., Shen J., Li M., Wang X., Ma L., Zhen C., Hou D., Liu E., Wang W., Wu G. (2017). Tuning Antiferromagnetic Exchange Interaction for Spontaneous Exchange Bias in MnNiSnSi System. APL Mater..

[40] Alexandrakis V., Aseguinolaza I.R., Decker P., Salomon S., Barandiarán J.M., Ludwig A., Chernenko V.A. (2018). Martensitic Transformation Hysteresis in Ni(Co)-Mn-Sn/MgO Metamagnetic Shape Memory Thin Films. Scr. Mater..

[41] Marcano N., Gómez Sal J.C., Espeso J.I., Fernández Barquín L., Paulsen C. (2007). Cluster-Glass Percolative Scenario in CeNi_1−x_Cu_x_ Studied by Very Low-Temperature Ac Susceptibility and Dc Magnetization. Phys. Rev. B Condens. Matter Mater. Phys..

[42] Alonso J., Fdez-Gubieda M.L., Sarmiento G., Chaboy J., Boada R., García Prieto A., Haskel D., Laguna-Marco M.A., Lang J.C., Meneghini C. (2012). Interfacial Magnetic Coupling between Fe Nanoparticles in Fe-Ag Granular Alloys. Nanotechnology.

[43] Djurberg C., Svedlindh P., Nordblad P. (1997). Dynamics of an Interacting Particle System: Evidence of Critical Slowing Down. Phys. Rev. Lett..

[44] Stanley H.E. (1999). Scaling, Universality, and Renormalization: Three Pillars of Modern Critical Phenomena. Rev. Mod. Phys..

[45] Hohenberg P.C., Halperin B.I. (1977). Theory of Dynamic Critical Phenomena. Rev. Mod. Phys..

